# *Plasmodium cynomolgi*: What Should We Know?

**DOI:** 10.3390/microorganisms12081607

**Published:** 2024-08-07

**Authors:** Fauzi Muh, Ariesta Erwina, Fadhila Fitriana, Jadidan Hada Syahada, Angga Dwi Cahya, Seongjun Choe, Hojong Jun, Triwibowo Ambar Garjito, Josephine Elizabeth Siregar, Jin-Hee Han

**Affiliations:** 1 Department of Epidemiology and Tropical Diseases, Faculty of Public Health, Universitas Diponegoro, Semarang 50275, Indonesia; fauzimuh010@lecturer.undip.ac.id (F.M.); ariestaerwina@alumni.undip.ac.id (A.E.); fadhilafitriana@alumni.undip.ac.id (F.F.); jadidanhada@alumni.undip.ac.id (J.H.S.); 2 Department of Environmental Health, Faculty of Public Health, Universitas Diponegoro, Semarang 50275, Indonesia; 13angga@alumni.undip.ac.id; 3 Department of Parasitology, School of Medicine, Chungbuk National University, Cheongju 28644, Republic of Korea; vetazmo@gmail.com; 4 Department of Environmental Biology and Tropical Medicine, School of Medicine, Kangwon National University, Chuncheon 24341, Republic of Korea; goodseed87@gmail.com; 5 Vector-Borne and Zoonotic Research Group, Research Center for Public Health and Nutrition, National Research and Innovation Agency Indonesia, Salatiga 50721, Indonesia; triw018@brin.go.id; 6 Eijkman Research Center for Molecular Biology, National Research and Innovation Agency, Jalan Raya Bogor Km. 46, Cibinong, Bogor 16911, Indonesia; jose001@brin.go.id

**Keywords:** *P. cynomolgi*, zoonosis, malaria

## Abstract

Even though malaria has markedly reduced its global burden, it remains a serious threat to people living in or visiting malaria-endemic areas. The six *Plasmodium* species (*Plasmodium falciparum*, *Plasmodium vivax*, *Plasmodium malariae*, *Plasmodium ovale curtisi*, *Plasmodium ovale wallikeri* and *Plasmodium knowlesi*) are known to associate with human malaria by the *Anopheles* mosquito. Highlighting the dynamic nature of malaria transmission, the simian malaria parasite *Plasmodium cynomolgi* has recently been transferred to humans. The first human natural infection case of *P. cynomolgi* was confirmed in 2011, and the number of cases is gradually increasing. It is assumed that it was probably misdiagnosed as *P. vivax* in the past due to its similar morphological features and genome sequences. Comprehensive perspectives that encompass the relationships within the natural environment, including parasites, vectors, humans, and reservoir hosts (macaques), are required to understand this zoonotic malaria and prevent potential unknown risks to human health.

## 1. Introduction

In the late 19th century, over 30 different *Plasmodium* species had been discovered, affecting human and non-human primates [1,2]. While there was a previous dominant belief that the natural transmission of simian malaria parasites to humans was rare, it has been firmly established for a significant period that specific non-human primates can acquire malaria, and this infection can also impact humans [3]. Various simian malaria species, *P. cynomolgi*, *P. knowlesi*, *P. inui*, *P. simium*, and *P. brasilianum*, have been intentionally transmitted in experiments and have been found to cause infections in humans through mosquito vectors [4,5]. Among these species, *P. cynomolgi*, the prevailing malaria parasite detected in Old-World monkeys within Southeast Asia, is recognized for its natural ability to cause zoonotic infections in humans [1,6,7,8,9]. As of now, there is no recorded evidence of *P. cynomolgi* being naturally transmitted from one human to another. Therefore, it is crucial to understand that the natural reservoir hosts for *P. cynomolgi*, long-tailed (*Macaca fascicularis*) and pig-tailed (*Macaca nemestrina*) macaques, are now living in close proximity to the human population, meaning humans are at a higher risk of contracting a *P. cynomolgi* infection [1,10]. Furthermore, numerous mosquito species that play a role in transmitting other types of human malaria can also serve as vectors for *P. cynomolgi*. This situation could potentially give rise to concerns regarding the transmission of the parasite between different species in regions where both the natural hosts and humans share the same habitat [4]. The potential for non-human malaria to spread to humans is increasing, primarily due to deforestation, significant shifts in ecological conditions, the presence of suitable hosts and vectors, and adaptive alterations in the biology of parasites due to climate changes [11,12]. In numerous Southeast Asian nations like Thailand, cases of *P. cynomolgi* infections are commonly found in pig-tailed and long-tailed macaques [9].

Furthermore, there is a well-documented instance of *P. cynomolgi* malaria naturally transmitting to a human in eastern Malaysia [3]. Additional monitoring in Northern Sabah, Malaysia, and Western Cambodia found asymptomatic human infections of *P. cynomolgi*, but they were not very common [7]. A symptomatic *P. cynomolgi* infection was confirmed in an individual who had recently returned to Denmark from Southeast Asia [13]. In Thailand, nine malaria-infected patients were discovered to be co-infected with both hidden *P. cynomolgi* and other *Plasmodium* species during the diagnosis of malaria patients presenting symptoms [9]. Typical clinical signs of *P. cynomolgi* infections usually involve symptoms like other clinical features of malaria such as headaches, reduced appetite, muscle discomfort, and nausea [3]. These symptoms typically manifest only during episodes of fever, are of moderate intensity, and can be effectively managed with basic medications used for the antimalarial regimen for *P. vivax*. [14]. The most notable physical observations consist of an enlarged spleen and liver [8]. *P. cynomolgi*, a simian malaria parasite, has emerged as a recent contributor to human malaria cases and continues to pose public health concerns in specific regions. In this review, we assess the updated literature on various aspects of *P. cynomolgi*, covering biology and genomes, epidemiology, its natural hosts and vectors, pathogenesis, and diagnostic approaches.

## 2. *Plasmodium cynomolgi* Biology

**Figure 1:**
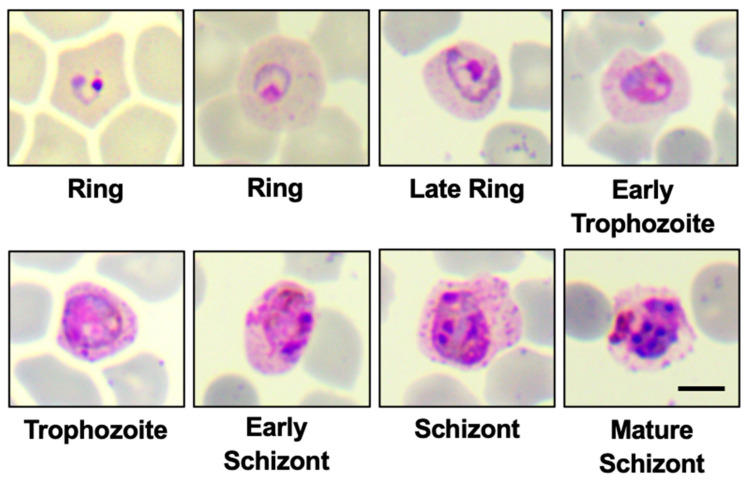
The morphology of *P. cynomolgi* K4 line is shown in each development stage cultured in 100% of rhesus macaque RBCs (Fauzi Muh et al., original unpublished data). Scale bar indicates 5 µm.

*P. cynomolgi* was initially studied and documented in Germany by Mayer in 1907. This study involved an imported *Macaca cynomolgus* from Java [15]. In 1937, H.W. Mulligan re-studied and re-described it as the Mulligan strain or M strain [16]. In the late 1950s, the *P. cynomolgi* Bastianielli strain or B strain was found by P.C.C. Garnham [17]. Other new *P. cynomolgi* strains (e.g., Berok, Cambodian, Gombak, Ceylonensis, and Smithsonian) were isolated from monkeys and mosquitoes [18,19,20,21,22,23]. Among studied strains in *Macaca mulatta*, the B strain typically exhibited higher mean parasitemia compared to the Mulligan (M) strain [24,25]. A study conducted earlier, between June 2013 and December 2017, at Kapit Hospital in Malaysia, involving 1047 blood samples from malaria patients, revealed that *P. cynomolgi* parasites were detected in the blood samples of two patients, making up approximately 1.5% to 4.7% of the total malaria parasite count [1]. The parasites *P. cynomolgi* had differentiated based on the morphological features of infected erythrocytes, including Schüffner’s stippling. Erythrocytes infected by the *P. cynomolgi* parasite undergo enlargement and can sometimes become distorted, with trophozoites of *P. cynomolgi* displaying single, double, or triple chromatin dots [1]. Research on *P. cynomolgi*’s M and B strains revealed liver incubation periods of 15 to 20 days and 16 to 37 days, respectively. The erythrocytic cycle takes 48 h, with a human prepatent period of 19 days. In *Macaca speciosa* and *Macaca mulatta*, commonly known hosts, the prepatent period spans from 7 to 16 days. [15]. Additionally, the asexual erythrocytic cycle of *P. cynomolgi* lasts for 48 h. [15,26]. In the early asexual stage of *P. cynomolgi*, infected red blood cells noticeably enlarge as the young parasite grows to almost half the size of the original host cell [25]. As the parasite undergoes its developmental stages, there is an augmentation in the prominence of Schüffner’s stippling and pigmentation. An observable resemblance emerges with *P. vivax* during the later trophozoite stage, where both trophozoites and schizonts exhibit the presence of Schüffner’s dots [15]. At maturity, *P. cynomolgi* produces an average of 16 merozoites, typically ranging from 14 to 20. The biology of *P. cynomolgi* closely resembles that of *non-Laverania* species, with shorter incubation and pre-patent periods compared to *P. falciparum* [8]. Obtained from different berok monkeys (K2, K3, and K4), the Berok K4 line culture was better than the others because it had a multiplication rate of two-fold to four-fold over more than five cycles (Figure 1) [25].

Multigene families are found to be very common in *P. cynomolgi* vs. *P. vivax*, and *P. cynomolgi* vs. *P. knowlesi* [27,28]. An analysis of 192 conserved ribosomal, translational, and transcriptional genes indicates that *P. cynomolgi* and *P. vivax* are closely related [28]. *P. cynomolgi* B strain (PcyB) genomes, isolated from monkeys in Malaysia, revealed 5722 genes with 90% of those genes (4613) being orthologs in *P. vivax* and *P. knowlesi* [28]. Meanwhile, the macaque-infected *P. cynomolgi* M strain (PcyM) has 966 new genes compared to PcyB [27]. In the development of a model for the study of other *Plasmodium* species, genes there were 214 genes found to be identical to both *P. cynomolgi* and *P. vivax*, and between *P. cynomolgi* and *P. knowlesi*, there were 100 genes found to be identical [27]. Consequently, the *P. cynomolgi* and *P. vivax* lineage typically contains a greater quantity of genes within multigene families compared to *P. knowlesi*, which indicates that repeated gene duplication occurred in the ancestor lines of *P. vivax* and *P. cynomolgi* or that some deletions may happen in *P. knowlesi*, such as the *vir*, *kir*, *SICAvar*, *Duffy binding protein* (*dbp*) and *reticulocyte binding protein* (*rbp*) genes [28]. Understanding the invasion biology of *Plasmodium* species is important. The gene families of *erythrocyte binding-like* (*ebl*) and *reticulocyte binding-like* (*rbl*) gene families are found to encode the parasite ligands required for successful invasion into red blood cells [29,30]. DBPs, molecules that interact with the Duffy antigen receptor for chemokines (DARC) found on the surface of both human and monkey erythrocytes, make up one of the *ebl* genes that encode the EBL ligands. *P. cynomolgi* has three *ebl* genes (*dbp1*, *dbp2*, *ebp*), in contrast to *P. vivax* and *P. knowlesi*, which have two (*dbp* and *ebp*) and three (*dbp-α*, *dbp-β*, *and dbp-γ*) genes, respectively. The *dbp* genes are thought to be the important ligands that invade the host’s red blood cells. The presence of more than one *dbp* in *P. cynomolgi* and *P. knowlesi* can also be thought to be responsible for infecting both human and monkey erythrocytes [31]. *P. cynomolgi* from different strains (Berok, Gombak, Cambodian, Rossan, Cylonesis, Smithsonian, B and M strain) showed 92% DNA identity or two very similar *dbp* genes [31]. The divergence of *rbp* genes has been thought to be involved in the species-specific erythrocyte invasion mechanism in different *Plasmodium* species [28]. The *rbl* genes encode large ligand proteins that exist on the apical membrane of invasive merozoites, such as *rbp* genes [32]. The variation in *rbp* genes has been shown between interspecies of *P. cynomolgi* strains, such as *rbp1b*, which exists in Berok and Gombak strains but is absent in the M/B, Rossan, Smithsonian, Ceylon, Langur and Cambodian strains [28,31]. Furthermore, *rbp2a* was absent in the Gombak and Berok strains. Thus, it is thought that the presence or absence of *rbp1b* or *rbp2a* compensate for each other when infecting the host’s red blood cells [31]. In the *P. cynomolgi* B strain, approximately 256 *pir* (plasmodium-interspersed repeat) superfamily or *cyir* (cynomolgi-interspersed repeat genes) are found [28]. Whilst in the *P. cynomolgi* M strain, a total of 1373 *cyir* genes are found [27]. *Cyir* genes are thought to have function related to immune evasion or antigenic variation [28,33,34,35]. The high variability of the genome in *P. cynomolgi* could be affected by natural host adaptation.

## 3. Natural Hosts and Vectors of *P. cynomolgi*

One study that inspected blood samples taken from *Macaca* monkeys in SEA countries mainly from Malaysia, found that, in its natural condition, the infections of *P. cynomolgi* in *Macaca* monkeys have been documented both as single infections and in combination with other simian malaria parasites (e.g., *P. vivax*, *P. inui*, *P. coatneyi*, and *P. fieldi*) [8]. Regarding the existence of *P. cynomolgi* in great apes, such as the orangutan (*Pongo pygmaeus*), one study found that the transmission of *P. cynomolgi* could possibly happen between the *Macaca* genera and the orangutan in Kalimantan Indonesia, whilst another study found that the orangutan is not a host of *P. cynomolgi* [36]. A systematic review of the prevalence of simian malaria in Malaysia, collected from seven studies conducted between 2000 and 2021, examined blood samples from *Macaca* genus and described that, from four studies, *P. cynomolgi* was commonly found in the *Macaca* species in Malaysia with an average prevalence of 33.05%. Studies also describe the type of infection among macaques in Malaysia. *P. cynomolgi* is commonly found in mono-infection and mix-infection with *P. inui* (dual infection), with *P. knowlesi* and *P. coatneyi* (triple infection), and with *P. knowlesi*, *P. coatneyi*, and *P. inui* (quadruple infection) [37]. Extensive research on wild long-tailed macaques (*M. fascicularis*) and pig-tailed macaques (*M. nemestrina*) in the region revealed that these species are hosts of a total of six simian malaria parasites, including *P. cynomolgi*, *P. coatneyi*, *P. fieldi*, *P. inui*, *P. knowlesi*, and *P. simiovale* (found in *Macaca sinica*) [15]. This discovery came after a large focus on humans [12,38]. *P. inui*, *P. knowlesi*, and *P. cynomolgi* were the three most common parasites among the 108 macaques studied (82%, 78% and 56%, respectively). In addition to *P. knowlesi*, *P. inui* and *P. cynomolgi* are two other simian parasites with zoonotic potential that have been demonstrated by unintentional and deliberate infections [1,15]. A study in the Philippines examined blood samples from 40 wild *Macaca fascicularis* (long-tailed macaque), as the natural host of *P. cynomolgi*, and described the prevalence of *Macaca fascicularis* monkeys infected by *P. cynomolgi* as 23.2% [39]. Another study describing the distribution of infected *M. fascicularis* with *P. cynomolgi* in countries of Southeast Asia (the Philippines, Indonesia, Cambodia, Singapore, and Laos) stated that *P. cynomolgi* was the most widespread parasite among all the sample populations with a prevalence of 53.3% [10].

The successful transmission of zoonotic malaria largely hinges on the ecological behavior and geographical prevalence of capable vectors. The changes in the habitat of vectors may have altered the bio-ecology of *Anopheles* mosquitoes [40,41]. The increase in zoonotic malaria is due to large deforestation required for changes to agriculture and human settlements, causing the mosquito vectors to live in close proximity to the host, both humans and macaque monkeys [42]. A change in land use, occupation, and settlements is often associated with proximity to infected vectors. Furthermore, mosquitoes that readily feed on humans and macaque monkeys harboring the parasites should live in a shared habitat with the reservoir hosts and the humans in order to cause the infection in the human population [43]. *P. cynomolgi* is mostly transmitted by *Anopheles leucosphyrus* subgroup mosquitoes [44]. A previous study conducted in seven states in Peninsular Malaysia revealed the infection of *P. cynomolgi* in *Anopheles introlatus*, and *An. Latens* [45]. The mosquito vectors can be infected by mono-*P. cynomolgi* infection or together with *P. inui* and *P. fieldi* [45]. In Sabah and Sarawak Malaysia, *An. balabacensis* were found to be infected with both single *P. cynomolgi* or together with *P. inui*, *P. knowlesi*, or *P. fieldi*. The *P. cynomolgi* that infects mosquitos, monkeys and humans showed the identity of nucleotide as 99.7–100%. This means that *P. cynomolgi* has a close relationship to those three isolates, monkey–human–mosquito, concluding that habitat sharing is the main factor of successful of *P. cynomolgi* transmission to the human population [46,47]. 

An investigation of the first natural *P. cynomolgi* infection by the Malaysian Vector Borne Disease Control Program found that the predominant mosquito within the patient’s housing area was *Anopheles cracens* [3]. In Thailand, *An. introlatus* has been shown to possess the *P. cynomolgi* in the salivary gland. *An. introlatus* is thought to be a responsible vector of *P. cynomolgi* in the Southern part of Thailand [9,48]. A study from Vietnam found that *P. cynomolgi* can be detected together with other simian malaria parasites in the primary mosquito vectors, *An. dirus* and *An. Minimus* [49]. This evidence can be a potential threat that the vector might be able to transmit to the human population, similar to *P. knowlesi* [50,51]. Furthermore, there was a study comparing the susceptibility of the *P. cynomolgi* B strain infection in *An. dirus*, *An. takasagoensis*, *An. maculatus*, and *An. philipinensis*. *An. dirus* showed the highest infectivity of *P. cynomolgi*, whereas *An. philipinensis* was shown to be the least susceptible [52].

## 4. Epidemiology

**Figure 2:**
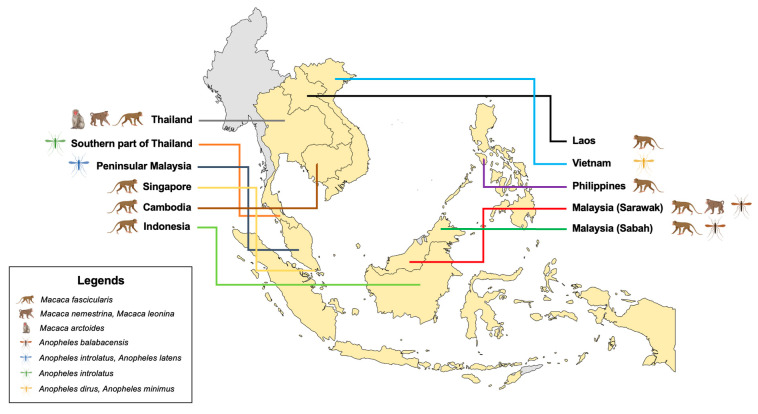
The geographical distribution of natural hosts and vectors of *P. cynomolgi*.

Transmission of *P. cynomolgi* through mosquito bites has been reported to be geographically spreading around Southeast Asia, with one case imported to Denmark (Figure 2 and Table 1). It is estimated that around 0–1.4% of people are infected with *P. cynomolgi* globally. Several factors that are associated with the spread of *P. cynomolgi* in natural infection are the presence of suitable vectors and non-primate hosts in a shared habitat, globalization, climate change, and deforestation [8]. *P. cynomolgi* infections usually appeared along with *P. falciparum* or *P. vivax* in the blood samples. *P. cynomolgi* may have the same Anopheline vectors or may have other vectors with a comparable zoophilic and anthropophilic tendency [1,15]. The existence of vectors plays an important role in the successful zoonotic malaria transmission of *P. cynomolgi*. It significantly relies on the bionomics and geographic distribution of the vectors, as well as the natural hosts of the parasite. Living in close proximity to hosts, particularly people working as farmers or in agriculture near the forests, or tourists traveling to a macaque endemic area, have a high risk of being exposed to *P. cynomolgi*. In a meta-analysis and systematic review of human *P. cynomolgi* infection cases in Malaysia (*n* = 8), a high proportion of natural hosts (macaque) was found to be infected with *P. cynomolgi*, accounting for 37.42% [53]. The existence of macaque (commonly pig-tailed and long-tailed species) infected with *P. cynomolgi* and other simian malaria parasites in Thailand, mainly in the southern region of the country, has been proven in a previous study [54]. The occurrence of transmission between humans and hosts is suspected to have happened because most of the patients infected with *P. cynomolgi* share the same environment with the host and mosquito. Patients co-infected with *P. cynomolgi*, *P. knowlesi*, and *P. vivax*, from a study in the Yala Province, had a history of living in a neighborhood surrounded by a group of domesticated pig-tailed and long-tailed macaques [9]. A study in Malaysia investigated the infection of simian malaria parasites among indigenous communities living in the forest fringe and found a mixed infection of *P. cynomolgi* with *P. inui*. These indigenous people were at high risk of simian malaria because of the high chance of exposure to monkeys, as the natural host, and mosquito bites [11].

## 5. Clinical Presentation

Malaria infections resulting from *P. cynomolgi* manifest numerous symptoms that overlap with those caused by other malaria species. Nevertheless, different strains of this parasite show some slight differences in their clinical signs. The striking feature of deliberately induced *P. cynomolgi* malaria infections in humans was the clear presence of substantial clinical symptoms, even when the level of parasites was low. These symptoms followed a sequence of cephalgia, anorexia, myalgia, and nausea [56]. Importantly, they typically occurred only during fever or febrile, were of moderate severity, and were easily treatable with antimalarial regimens for *P. vivax* [14]. Additionally, common physical findings included a splenomegaly and a hepatomegaly [8].

*P. cynomolgi* infections are frequently found in macaque monkeys, including *Macaca fascicularis* (long-tailed macaque), *Macaca nemestrina*, and *Macaca leonina* (pig-tailed macaque) (Figure 2). In Java, *P. cynomolgi* was first discovered by Halberstadter and von Prowazek in 1907 from long-tailed macaques (*M. fascicularis*). Similar to *P. vivax*, *P. cynomolgi* was reported to have recurrent relapses in rhesus monkeys. This exoerythrocytic source was later identified as dormant “hypnozoites” in the liver. As a result, it was used as the animal model for recurrent malaria. *P. cynomolgi* first infected humans accidentally with the *P. cynomolgi* B strain in 1960. This infection was later acquired naturally by humans in Southeast Asia from various macaque monkeys [57,58,59,60,61].

The first known case of naturally acquired *P. cynomolgi* malaria in humans was reported in 2011. The patient was a 39-year-old woman from the east coast of Peninsular Malaysia with no previous history of malaria who did not travel to any other malaria-endemic areas. The clinical symptoms of the patient were non-specific and mimicked a flu-like syndrome with a febrile condition. The patient took oral chloroquine for medication and recovered within a week.

Additional symptoms, including muscle pain, general malaise, headache, fever, and abdominal pain were reported by a traveler who had returned from a Southeast Asian country. A blood test revealed elevated C reactive protein, S-alanine aminotransferase (S-ALAT), thrombocytopenia, and low platelet levels [13]. The prepatent period, defined as the duration between infection and the onset of symptoms, ranges from 7 to 16 days. The incubation period, which is the time between infection and the development of the disease, ranges from 15 to 20 days, with some differences seen in different *P. cynomolgi* strains. On the other hand, patients infected with *P. cynomolgi* typically experience, at worst, mild and non-life-threatening symptoms [3,7,13,55]. An experimental study described that both the *P. cynomolgi* M and B strains had similar significant symptoms, which included high fever, headaches, loss of appetite, muscle pain, and nausea. However, there were differences in how long these symptoms lasted, how often fever episodes occurred, and the degree of spleen enlargement. Individuals infected with the M strain exhibited prolonged symptoms, a higher incidence of tertian fever, and an increased likelihood of developing splenomegaly when compared to those infected with the B strain. Additionally, only those intentionally infected with the M strain reported experiencing chills and vomiting [56].

## 6. *P. cynomolgi* Confirmation and Diagnosis

*P. cynomolgi* exhibits phenotypic and phylogenetic resemblances to *P. vivax*, posing difficulties in differentiation between the two when examining blood smears using routine microscopy. Often, routine microscopy can lead to the misdiagnosis of *P. knowlesi as P. malariae* and *P. cynomolgi* as *P. vivax* [62]. In such situations, it becomes essential to precisely assess and comprehend the prevalence and transmission patterns of non-human *Plasmodium* species, particularly *P. cynomolgi*, within human populations using a more sensitive diagnostic tool [3,6,63]. Molecular methods are the most accurate diagnostic test for morphologically identical species [64]. The first case of natural infection of *P. cynomolgi* in humans was confirmed by molecular detection, using nested PCR. The sequencing result of 785 nucleotides showed that 99.9% of the genes were similar to the *P. cynomolgi* M-strain from Malaysia [3,15]. A recently developed test to confirm the presence of *P. cynomolgi* used a combined lateral flow with a recombinase polymerase amplification (RPA-LFD) [6]. This assay uses the designed *18S rRNA* primers. From a total of 30 *Plasmodium*-positive blood samples from wild macaques, nested PCR detected positivity in 11 out of these samples, and 9 positives were detected by RPA-LFD assay. Of the 19 negative samples by nested PCR, RPA-LFD assay showed 18 true negatives. The limit of detection (LoD) of this assay also showed 22.14 copies/μL, which was said to be 10 times greater than qPCR or RPA-AGE (Agarose Gel Electrophoresis) assay. It is concluded that the latest developed test has given 81.82% sensitivity and 94.74% specificity in detecting the target DNA of *P. cynomolgi* [6]. However, this assay may require specialized techniques and require a longer time for sample preparation. This technique may not be suitable for use in a resource-limited setting. Another test, qPCR, is also performed to confirm the *P. cynomolgi* targeting the *18S rRNA* gene. The LoD observed was 0.075 ng/μL. However, using the suspected clinical isolates (*n* = 250 human blood samples), the qPCR was not able to confirm the presence of *P. cynomolgi* [63]. The inability to detect *P. cynomolgi* in the clinical samples may be due to the low DNA content in the samples, or this may be the true negative result of *P. cynomolgi.* Thus, a large number of clinical samples may be required to validate the use of qPCR for the detection of the presence of *P. cynomolgi* DNA. Furthermore, loop-mediated isothermal amplification (LAMP) is an alternative assay that reduces the downsides of a PCR diagnosis-based assay. The LAMP assay is employed with a focus on species-specific targeting of the mitochondrial genes [65,66,67,68,69,70,71,72]. Studies have indicated that LAMP exhibits greater sensitivity and specificity compared to ELISA and microscopy, with a reported accuracy of 95.6% and 100%, respectively [73].

## 7. *P. cynomolgi* for *P. vivax* Malaria Research

*P. cynomolgi* is a species genetically related to *P. vivax*, which is one of the malaria parasite species in humans. *P. cynomolgi* naturally infects monkeys, especially long-tailed macaques (Figure 2). Although *P. cynomolgi* usually does not cause serious illness in humans, this parasite has many similarities with *P. vivax*, especially relating to phenotype, biology, and genetic characteristics [27,74,75]. The lack of a long-term in vitro culture method for *P. vivax* has severely limited our understanding of the *P. vivax* invasion biology and drug and vaccine development. However, the putative *P. vivax* drug-resistance marker *mdr1* Y976F was investigated by using *P. cynomolgi* Berok in an in vitro culture model. By conducting genetic manipulations in *P. cynomolgi* and observing the ensuing alterations in its characteristics, this model is able to identify the specific impact of the Y976F mutation within the *Pcymdr1* gene on drug sensitivity [75].

*P. vivax* merozoites predominantly invade human reticulocytes, thereby limiting the development of an in vitro culture. The inability to establish a continuous culture system for *P. vivax* affects the development of a growth inhibition assay (GIA). To address this limitation, a previous study utilized a surrogate species like *P. cynomolgi* as a model for *P. vivax* [76]. *P. cynomolgi* is commonly used in monkey studies for drug discovery and understanding the biological characteristics. The capacity to infect human reticulocytes using *P. cynomolgi* creates valuable opportunities for studying invasion mechanisms and changes in red blood cells. A previous study showed success in growing the blood stages of two *P. cynomolgi* strains, particularly the Berok strain. Initially, from a wild *M. nemestrina* in Malaysia in the 1960s, the Berok strain was maintained by blood or sporozoite inoculation in monkeys. This strain was not cloned, suggesting it might contain various parasite types that could vary during infection or across hosts [21]. It is unclear if Berok parasites from different monkeys differ genetically or as variations within a group. Addressing initial setbacks persistently could likely lead to cultivating other *P. cynomolgi* lines or macaque parasite species. The study highlighted a valuable surrogate for *P. vivax* to expand research possibilities with lab-cultured parasites. Research findings confirm that lab-grown Berok K4 parasites resemble those from monkeys and maintain infectivity to produce infective sporozoites in mosquitoes. This study has shown the possible use of a *P. cynomolgi* model for *P. vivax* research to understand the morphology, characteristics, and behavior [25]. The previous study has shown that *P. cynomolgi* in in vitro culture was totally restricted to human reticulocytes. Meanwhile, the in vitro cultures of *P. cynomolgi* using monkey RBCs were not restricted to only reticulocytes [77]. The *P. cynomolgi* has two invasion pathways (DARC-dependent and independent) to invade the rhesus macaques [78]. However, the invasion into human RBCs by *P. cynomolgi* depends on the DARC-dependent pathway [77]. It serves as a great model to assess vaccines before clinical trials, contributing significantly to developing new strategies to control the widespread and challenging *P. vivax* [77].

## 8. Treatment

The importance of malaria management lies in preventing the transmission of the disease and decreasing the immediate risk to the host. One aspect of this management is treating malaria patients with specific medications. Currently, there is no single therapy that can eradicate *P. cynomolgi* at each respective lifecycle stage. Some studies reported that *P. cynomolgi*-infected patients were treated with a combination of existing antimalarial drugs, such as atovaquone plus proguanil or chloroquine plus primaquine. Moreover, another study found that *P. cynomolgi* co-infected with *P. falciparum* patients were treated using artesunate plus mefloquine [9,13,14].

The significant morphological and biological characteristics of both *P. vivax* and *P. cynomolgi* are the dormant liver stages (hypnozoites), which are responsible for relapses due to their reactivation within several weeks to years after the initial infection. It is essential to prevent relapses of *P. cynomolgi* through safer radical curative compounds that efficiently kill hypnozoites. Similar to *P. vivax*, previous research has identified primaquine as a potential treatment for *P. cynomolgi* infections against hypnozoites. However, primaquine can cause acute hemolytic anemia in malaria patients with glucose-6-phospate dehydrogenase (G6PD) deficiency [4]. Additionally, the CDC’s recommendation of a primaquine treatment schedule of 30 mg/day for 14 days for non-G6PD-deficient patients can lead to primaquine resistance due to limited patient compliance. A previous study was conducted to identify a new potent non-8-aminoquinoline compound that efficiently kills the early developmental forms of hypnozoites in vitro using a drug assay. The result shows that the activity of KAF156 was limited to schizont; meanwhile, KAI 407 showed activity against both liver stages schizonts and hypnozoites forms, like primaquine [79]. Another study found that MMV019721, which is an acetyl-CoA synthetase inhibitor that affects histone acetylation, selectively kills *P. vivax* and *P. cynomolgi* hypnozoites [80].

## Figures and Tables

**Table 1:** Natural human infection of *P. cynomolgi*.

Study Locations	Samples (*n*)	Human Malaria Positive (*n*)	Type of Infection	
			Mono-Infection (*n*)	Mixed Infection (*n*)
Thailand (2007–2016) (Putaporntip, C. et al., 2021 [9])	1359	1180	0	*P. cynomolgi* + *P. vivax* (7); *P. cynomolgi* + *P. falciparum* (1); *P. cynomolgi* + *P. falciparum* + *P. vivax* + *P. knowlesi* (1)
Malaysia (first case 2011) (Ta, T. H. et al., 2014 [3])	-	1	1	0
Malaysia (2011–2014) (Yap, N. J. et al., 2021 [11])	288	90	9	0
Cambodia (2013–2016) (Imwong, M. et al., 2019 [7])	14,732	1361	11	*P. cynomolgi* + *P. vivax* (2)
Malaysia (2013–2017) (Raja, T. N. et al., 2020 [1])	-	1047	0	*P. cynomolgi* + *P. knowlesi* (6)
Malaysia (2015) (Grignard, L. et al., 2019 [55])	876	54	2	0
Denmark (2018) (Hartmeyer, G. N. et al., 2019 [13])	-	1	1	0
Thailand (2008–2016) (Putaporntip, C. et al., 2010 [54])	5271	4195	2	*P. cynomolgi* + *P. vivax* (2); *P. cynomolgi* + *P. vivax* (1); *P. cynomolgi* + *P. vivax* (1); *P. cynomolgi* + *P. vivax* (3); *P. cynomolgi* + *P. vivax* (8); *P. cynomolgi* + *P. falciparum* (2); *P. cynomolgi* + *P. knowlesi* (2)
Thailand (2015) (Sai-ngam, P. et al., 2022 [14])	3	3	2	*P. cynomolgi* + *P. vivax* (1)

## Data Availability

All data that support this study are available upon request to the corresponding author.
